# Association between dentists’ awareness of antimicrobial resistance and selection of oral third-generation cephalosporins: a cross-sectional study

**DOI:** 10.1017/ash.2026.10775

**Published:** 2026-07-27

**Authors:** Tatsuya Tai, Takahiro Motoki, Naohiro Kurokawa, Sayaka Yamashita, Kazunori Yamaguchi, Hiroaki Tanaka, Shinji Kosaka, Yuuichi Muraki

**Affiliations:** 1 Kagawa University Hospital: Kagawa Daigaku Igakubu Fuzoku Byoin, Japan; 2 Department of Pharmacy, Kagawa University Hospital, Mikicho, Japan; 3 Department of Safety Management, Kagawa University Hospital, Mikicho, Japan; 4 Laboratory of Clinical Pharmacoepidemiology, Kyoto Pharmaceutical University, Kyoto, Japan

## Abstract

**Objective:**

Antimicrobial resistance (AMR) is a global public health concern. In Japan, outpatient dental practice frequently involves antimicrobial prescriptions, with oral third-generation cephalosporins prescribed more often than the guideline-recommended amoxicillin. We examined the association between dentists’ multidimensional AMR-related attitudes and selection of oral third-generation cephalosporins.

**Design:**

Cross-sectional secondary analysis of a questionnaire survey.

**Participants:**

The questionnaire survey was administered to 406 dentists affiliated with the Kagawa Dental Association (November 2024–January 2025), and 111 responses were analyzed (response rate, 27.3%).

**Methods:**

The primary outcome was self-reported selection of oral third-generation cephalosporins. AMR attitudes were quantified as overall and domain-specific scores for sense of crisis, self-efficacy, and role awareness. Multivariable logistic regression analysis was performed, adjusting for years of clinical experience, sex, post-treatment prophylactic antimicrobial use, and practice type (in-house vs. external pharmacy dispensing). Domain-specific scores were analyzed separately to avoid multicollinearity.

**Results:**

High overall AMR attitude scores were associated with a low likelihood of selecting third-generation cephalosporins (odds ratio [OR], 0.82; 95% confidence interval [CI], 0.72–0.92). Self-efficacy (OR, 0.50; 95% CI, 0.34–0.73) and role awareness (OR, 0.59; 95% CI, 0.42–0.84) showed strong negative associations. Post-I treatment prophylactic antimicrobial use and practice type involving external pharmacy dispensing were positively (OR, 2.69; 95% CI, 1.07–6.75) and negatively (OR, 0.34; 95% CI, 0.15–0.77) associated with selection of third-generation cephalosporins, respectively.

**Conclusions:**

Dentists’ AMR-related attitudes, particularly self-efficacy and professional role awareness, were strongly associated with antimicrobial prescription choices. Interventions targeting these factors may promote appropriate antimicrobial use in dental practice.

## Introduction

Antimicrobial resistance (AMR) is a serious global public health concern. In Japan, efforts to reduce antimicrobial use and the incidence of resistant organisms have been promoted under the “National Action Plan on AMR.”^1,2^ Under the Japanese national healthcare system, outpatient antimicrobial prescriptions may be dispensed either within dental clinics or community pharmacies, reflecting different degrees of separation between prescription and dispensing processes. The dental field is predominantly characterized by outpatient care, in which antimicrobials are frequently prescribed and account for approximately 10% of the total antimicrobial consumption in Japan. However, in Japanese dental practice, oral third-generation cephalosporins are used more frequently than amoxicillin, which is recommended as the first-line agent, and this prescribing pattern has been noted to diverge from international guidelines.^3–5^

To address this discrepancy, it is important to focus not only on improving knowledge but also on the role of “attitudes,” including a sense of urgency regarding the AMR problem, self-efficacy in being able to prescribe in accordance with guidelines, and professional role awareness. Studies conducted overseas have shown that although educational interventions can improve knowledge, such improvements do not necessarily lead to sustained behavioral changes.^6^ In addition, domestic surveys have reported that while dentists demonstrate a high level of awareness of AMR, inappropriate or uniform prescribing practices remain common, suggesting a gap between knowledge and actual prescribing behavior.^7,8^

Recent systematic reviews have also identified dentists’ attitudes as factors that may influence their prescribing behavior.^9^ However, studies that quantitatively assess attitudes toward AMR and examine their association with specific antimicrobial choices remain limited, both domestically and internationally. In particular, the relationship between AMR-related attitudes and the selection of oral third-generation cephalosporins has not been sufficiently elucidated.

Therefore, we aimed to examine the association between dentists’ multidimensional awareness of AMR and the selection of oral third-generation cephalosporins by analyzing data from an existing questionnaire survey conducted for educational purposes.

## Methods

### Study design

This was a cross-sectional secondary analysis of questionnaire data collected for educational purposes.

### Participants and survey content

This study was a secondary analysis of data from a questionnaire survey conducted among dentists in the Department of Infection Control at Kagawa University Hospital. The survey was conducted between November 29, 2024, and January 18, 2025, and targeted 406 dentists affiliated with the Kagawa Dental Association. The questionnaire was distributed via Google Forms and fax through the Department of Pharmacy at Kagawa University Hospital, and the responses were collected voluntarily. During the survey period, 111 responses were obtained (response rate, 27.3%), all of which were included in the analysis.

The questionnaire was developed based on previous studies.^6^ ^–^ ^8^ ^,^ ^10^ The survey items included the selection of oral third-generation cephalosporins (binary variable), sex, years of clinical experience, purpose of antimicrobial prescription (including posttreatment prophylactic use), practice type categorized as in-house dispensing (medications dispensed within the dental clinic) or external pharmacy dispensing (prescriptions filled at community pharmacies under the separation of prescription and dispensing processes), and attitude related to AMR and appropriate antimicrobial use assessed using Likert scales.

### AMR attitude score

Items assessing attitudes toward AMR were quantified to calculate the overall AMR attitude scores. Based on previous studies, AMR-related attitudes were categorized into three domains: “sense of crisis,” “self-efficacy,” and “role awareness.”^6^

The “sense of crisis” domain comprised items reflecting perceptions of the seriousness of AMR and the importance of adherence to the guidelines. The “self-efficacy” domain included items related to confidence in being able to prescribe in accordance with the guidelines and the perceived ease of referring to such guidelines. The “role awareness” domain comprised items evaluating dentists’ sense of professional responsibility in reducing the risk of AMR.

### Outcome measures

The primary outcome was the selection of oral third-generation cephalosporins, defined as dentists’ self-reported selection of these agents.

AMR attitudes were quantified using overall and domain-specific scores, including a sense of crisis, self-efficacy, and role awareness. In addition to the primary analysis focusing on the overall score, domain-specific scores were examined separately to explore their individual associations with prescribing behavior.

### Statistical analysis

For the primary analysis, multivariable logistic regression analysis was performed, with the selection of oral third-generation cephalosporins as the dependent variable and the overall AMR attitude score as the main independent variable. Years of clinical experience, sex, use of prophylactic prescriptions, and practice type (in-house dispensing vs external pharmacy dispensing) were prespecified as covariates and were included in the models for adjustment. Because domain-specific attitude scores were likely to correlate with the overall score, exploratory analyses were performed, in which each domain score was entered separately into individual models, distinct from the primary analysis. The overall and domain-specific scores were not included in the same model to avoid multicollinearity.

For the sensitivity analysis, models that excluded cases involving prophylactic prescriptions were also fitted. A two-sided *P*-value of <.05 was considered statistically significant. All analyses were performed using R version 4.4.3 (R Foundation for Statistical Computing, Vienna, Austria). The model fit for each logistic regression analysis was assessed using likelihood ratio tests, and multicollinearity was examined using variance inflation factors. Because the aim of this study was to evaluate the independent association between AMR attitudes and prescription choices while accounting for potential confounders, only adjusted odds ratios (ORs) derived from multivariable analyses are presented as the primary results.

### Ethics statements

This secondary analysis was performed using existing questionnaire data that had been collected for educational purposes. Before the analysis, the researchers removed personal identifiers, including names, contact information, and facility names, from the free-text responses and anonymized the data. No new surveys or research interventions were conducted. For these reasons, the study was exempted from ethical review by the Ethics Review Board of Kagawa University (approval number: 2025-194).

## Results

### Participant characteristics

In total, 111 dentists were included in the analysis. The detailed participant characteristics and practice-related variables are shown in Table 1. The median number of years of clinical experience was 31.0 (25.0–38.5) years, and 55 dentists (49.5%) reported selecting oral third-generation cephalosporins. The distributions of the overall and domain-specific AMR attitude scores are summarized in Table 2.

**Table 1.** Participant characteristics Table 1 long description.

Characteristic	Overall
Participants, n	111
Male sex, n (%)	95 (85.6)
Years of clinical experience, median [interquartile range]	31.0 [25.0–38.5]
Oral third-generation cephalosporin use, n (%)	55 (49.5)
Posttreatment prophylactic antimicrobial use, n (%)	41 (36.9)
Practice type (external pharmacy dispensing), n (%)	52 (46.8)

For each variable, values are presented as numbers (percentages) or median (25th–75th percentile interquartile range), as applicable.

**Table 2.** Antimicrobial resistance attitude scores Table 2 long description.

Score	Median [interquartile range]
Overall AMR attitude score	30.0 [27.0–32.0]
Sense of crisis score	16.0 [14.0–16.0]
Self-efficacy score	7.0 [6.0–8.0]
Role awareness score	7.0 [6.0–8.0]

Values are presented as median (25th–75th percentile interquartile range). The overall AMR attitude score represents the sum of all attitude items. Domain-specific scores include a sense of crisis, self-efficacy, and role awareness.

AMR, antimicrobial resistance.

### Overall AMR attitude score and prescribing behavior

High overall AMR attitude scores were negatively associated with the selection of oral third-generation cephalosporins (OR, 0.82; 95% confidence interval [CI], 0.72–0.92; *P* = .001). In addition, the practice type involving external pharmacy dispensing was independently associated with a low likelihood of selecting oral third-generation cephalosporins (OR, 0.34; 95% CI, 0.15–0.77; *P* = .011). In contrast, years of clinical experience, sex, and use of posttreatment prophylactic antimicrobials were not significantly associated with prescription selection (Table 3).

**Table 3.** Factors associated with selection of oral third-generation cephalosporins: multivariable logistic regression analysis Table 3 long description.

Variable	Overall AMR attitude score	Sense of crisis score	Self-efficacy score	Role awareness score
AMR attitude score (per 1-point increase)	0.82 (0.72–0.92), 0.001	0.77 (0.61–0.96), 0.021	0.50 (0.34–0.73), <0.001	0.59 (0.42–0.84), 0.003
Years of clinical experience	1.01 (0.97–1.05), 0.62	1.01 (0.97–1.05), 0.58	1.00 (0.96–1.05), 0.89	1.01 (0.97–1.05), 0.67
Male sex	1.32 (0.44–3.94), 0.62	1.28 (0.42–3.89), 0.66	1.41 (0.44–4.48), 0.56	1.36 (0.43–4.28), 0.60
Posttreatment prophylactic antimicrobial use	1.88 (0.78–4.54), 0.16	1.94 (0.80–4.69), 0.14	2.69 (1.07–6.75), 0.036	1.97 (0.80–4.86), 0.14
Practice type (external pharmacy dispensing)	0.34 (0.15–0.77), 0.011	0.34 (0.16–0.77), 0.009	0.29 (0.13–0.69), 0.005	0.33 (0.15–0.75), 0.008
No. of participants	111	111	111	111

Values are adjusted odds ratios with 95% confidence intervals and *P*-values. The outcome was the selection of oral third-generation cephalosporins. Each model included years of clinical experience, sex, posttreatment prophylactic antimicrobial use, and practice type (external pharmacy dispensing). The overall and domain-specific antimicrobial resistance attitude scores were entered into separate models to avoid multicollinearity.

AMR, antimicrobial resistance.

### Domain-specific attitude scores and prescribing behavior

#### Sense of crisis score

A high sense of crisis score was significantly associated with a low likelihood of selecting oral third-generation cephalosporins (OR, 0.77; 95% CI, 0.61–0.96; *P* = .021). Practice type involving external pharmacy dispensing was also associated with prescription selection (OR, 0.34; 95% CI, 0.16–0.77; *P* = .009) (Table 3).

#### Self-efficacy score

High self-efficacy scores were strongly associated with a low likelihood of selecting oral third-generation cephalosporins (OR, 0.50; 95% CI, 0.34–0.73; *P* < .001). In contrast, the use of posttreatment prophylactic antimicrobials was positively associated with prescription selection (OR, 2.69; 95% CI, 1.07–6.75; *P* = .036). Practice type involving external pharmacy dispensing was negatively associated with prescription selection (OR, 0.29; 95% CI, 0.13–0.69; *P* = .005) (Table 3).

#### Role awareness score

High role awareness scores were significantly associated with a low likelihood of selecting oral third-generation cephalosporins (OR, 0.59; 95% CI, 0.42–0.84; *P* = .003). Practice type involving external pharmacy dispensing was similarly associated with prescription selection (OR, 0.33; 95% CI, 0.15–0.75; *P* = .008) (Table 3).

#### Model fit

The likelihood ratio tests were statistically significant for all multivariable logistic regression models. The variance inflation factors for all explanatory variables ranged from 1.0 to 1.1, indicating no evidence of problematic multicollinearity.

## Discussion

In this study, we assessed dentists’ attitudes toward AMR and examined their cross-sectional association with the selection of oral third-generation cephalosporins. A distinctive feature of this study is that it focused on a population of dentists who primarily prescribed antimicrobials in outpatient settings, allowing us to assess prescription choices in a context similar to real-world clinical practice. The results suggested that dentists with high overall AMR attitude scores had low odds of selecting oral third-generation cephalosporins. These findings indicate that heightened awareness of AMR may extend beyond knowledge or attitudes alone and be reflected in actual prescribing decisions, thereby contributing to behaviors aligned with appropriate antimicrobial use^11,12^.

In exploratory analyses according to domain, self-efficacy and professional role awareness showed strong associations with prescription selection. These findings suggest that, in addition to awareness of the AMR problem itself, self-efficacy in being able to prescribe in accordance with the guidelines and recognition of professional responsibility for appropriate antimicrobial use may be associated with prescription decisions. Previous studies have shown that psychological and behavioral factors such as attitudes and self-efficacy are more influential than knowledge levels alone in antimicrobial stewardship, and the results of this study are consistent with these findings.^13,14^

Although the sense of crisis score was also associated with prescription selection, the strength of this association was limited compared with that of self-efficacy and role awareness. These findings suggest that while a sense of urgency regarding AMR is an important prerequisite for behavioral change, perceptions of being able to act (self-efficacy) and recognition that such actions are part of one’s professional role may be critical in translating awareness into actual prescribing behavior.^15,16^ In other words, a sense of crisis alone may be insufficient to induce behavioral change, and psychological factors that support action may mediate this process.

In addition, the type of practice involving external pharmacy dispensing was significantly associated with the selection of oral third-generation cephalosporins. In the Japanese healthcare system, external pharmacy dispensing reflects a great separation between prescription and dispensing processes in outpatient dental practice. In systems involving outpatient prescriptions, pharmacists may play a role in reviewing prescription content, providing information, and offering feedback. This suggests that institutional and environmental factors, together with individual dentists’ attitudes, may influence prescribing behavior. Although causality cannot be inferred from this cross-sectional analysis, previous studies have revealed that external review mechanisms can contribute to appropriate antimicrobial use. The findings of this study extend this line of evidence.^17^

This study has several limitations. First, because of its cross-sectional design, causal relationships between attitudes and prescribing behaviors cannot be directly inferred, and the possibility of reverse causation cannot be excluded. Second, the outcome was based on whether a specific antimicrobial was selected for prescription and did not assess the prescription frequency, appropriateness of indications, or inappropriate prescription. Third, the generalizability of the findings may be limited because the study population was limited to dentists from a specific geographic region.

Despite these limitations, this study provides evidence of the associations between the multidimensional aspects of AMR-related attitudes and antimicrobial selection in dental settings. Although causal inferences cannot be drawn from this cross-sectional analysis, the strong associations observed between self-efficacy and professional role awareness and prescribing behavior suggest that future AMR interventions may need to move beyond the provision of knowledge or the promotion of a sense of crisis alone and instead place emphasis on psychological and professional factors that support appropriate prescribing behavior. Future studies using longitudinal designs or interventional approaches are warranted to examine whether educational and institutional interventions aimed at strengthening self-efficacy and role awareness can influence antimicrobial prescribing behaviors in dental practice.

In this study, we conducted a multidimensional assessment of dentists’ attitudes toward AMR and examined its association with the selection of oral third-generation cephalosporins. Our findings showed that dentists with high overall AMR-related attitude scores were unlikely to select broad-spectrum antimicrobials. Particularly, components such as self-efficacy and professional role awareness were strongly associated with prescribing behavior.

These results suggest that promoting appropriate antimicrobial use in dental practice requires not only the provision of knowledge and raising risk awareness but also interventions that enhance dentists’ self-efficacy in prescribing in accordance with the guidelines and their recognition of professional responsibility. This study provides a foundation for developing educational and institutional interventions for AMR stewardship in the field of dentistry.

## Data Availability

Data are available upon request and subject to privacy, ethics, and other restrictions.

## Table 1. Long description

A table summarizing characteristics of 111 dentists, including clinical experience, antimicrobial use, and practice type. The table has two columns: Characteristic and Overall. The characteristics listed are Participants, Male sex, Years of clinical experience, Oral third-generation cephalosporin use, Posttreatment prophylactic antimicrobial use, and Practice type. The overall values are 111 participants, 95 male (85.6 percent), a median of 31.0 years of clinical experience with an interquartile range of 25.0 to 38.5 years, 55 using oral third-generation cephalosporins (49.5 percent), 41 using posttreatment prophylactic antimicrobials (36.9 percent), and 52 engaged in external pharmacy dispensing (46.8 percent).

Navigate back to Table 1..

## Table 2. Long description

A table with four rows and two columns. The columns are labeled ‘Score’ and ‘Median [interquartile range]’. The rows are labeled with different scores: Overall AMR attitude score, Sense of crisis score, Self-efficacy score, and Role awareness score. Each row contains a score value and a median value with an interquartile range. Row 1: Overall AMR attitude score, 30.0; 27.0-32.0. Row 2: Sense of crisis score, 16.0; 14.0-16.0. Row 3: Self-efficacy score, 7.0; 6.0-8.0. Row 4: Role awareness score, 7.0; 6.0-8.0.

Navigate back to Table 2..

## Table 3. Long description

A table with four columns and five rows, plus a header row. The columns are labeled Variable, Overall AMR attitude score, Sense of crisis score, Self-efficacy score, and Role awareness score. The rows list different variables and their associated scores and confidence intervals. Row 1: AMR attitude score (per 1-point increase), 0.82 (0.72-0.92), 0.001, 0.77 (0.61-0.96), 0.021, 0.50 (0.34-0.73), <0.001, 0.59 (0.42-0.84), 0.003. Row 2: Years of clinical experience, 1.01 (0.97-1.05), 0.62, 1.01 (0.97-1.05), 0.58, 1.00 (0.96-1.05), 0.89, 1.01 (0.97-1.05), 0.67. Row 3: Male sex, 1.32 (0.44-3.94), 0.62, 1.28 (0.42-3.89), 0.66, 1.41 (0.44-4.48), 0.56, 1.36 (0.43-4.28), 0.60. Row 4: Posttreatment prophylactic antimicrobial use, 1.88 (0.78-4.54), 0.16, 1.94 (0.80-4.69), 0.14, 2.69 (1.07-6.75), 0.036, 1.97 (0.80-4.86), 0.14. Row 5: Practice type (external pharmacy dispensing), 0.34 (0.15-0.77), 0.011, 0.34 (0.16-0.77), 0.009, 0.29 (0.13-0.69), 0.005, 0.33 (0.15-0.75), 0.008. The table includes the number of participants as 111 for each variable.

Navigate back to Table 3..
